# Mobile Health for First Nations Populations: Systematic Review

**DOI:** 10.2196/14877

**Published:** 2019-10-07

**Authors:** Georgina R Hobson, Liam J Caffery, Maike Neuhaus, Danette H Langbecker

**Affiliations:** 1 Centre for Online Health Centre for Health Services Research The University of Queensland Woolloongabba Australia

**Keywords:** mHealth, mobile health, indigenous, First Nations, aboriginal, humans, systematic review

## Abstract

**Background:**

The ubiquitous presence and functionality of mobile devices offers the potential for mobile health (mHealth) to create equitable health opportunities. While mHealth is used among First Nations populations to respond to health challenges, the characteristics, uptake, and effectiveness of these interventions are unclear.

**Objective:**

This review aimed to identify the characteristics of mHealth interventions (eg, study locations, health topic, and modality) evaluated with First Nations populations and to summarize the outcomes reported for intervention use, user perspectives including cultural responsiveness, and clinical effectiveness. In addition, the review sought to identify the presence of First Nations expertise in the design and evaluation of mHealth interventions with First Nations populations.

**Methods:**

The methods of this systematic review were detailed in a registered protocol with the International Prospective Register of Systematic Reviews (PROSPERO, CRD42019123276). Systematic searches of peer-reviewed, scientific papers were conducted across 7 databases in October 2018. Eligible studies had a primary focus on mHealth interventions with experimental or quasi-experimental design to respond to a health challenge with First Nations people from Canada, Australia, New Zealand, and the United States. Two authors independently screened records for eligibility and assessed risk of bias using the Joanna Briggs Institute checklists. Data were synthesized narratively owing to the mix of study designs, interventions, and outcomes. The review was reported in accordance with the Preferred Reporting Items for Systematic Reviews and Meta-Analyses statement.

**Results:**

Searches yielded 1053 unique records, after review and screening, 13 studies (5 randomized controlled trials and 8 quasi-experimental designs) were included in the final analysis. Studies were conducted in Australia (n=9), the United States (n=2), and New Zealand (n=2). The most common health challenge addressed was mental health and suicide (n=5). Intervention modalities included text messaging (n=5), apps (n=4), multimedia messaging (n=1), tablet software (n=1), or a combination of short messaging service (SMS) and apps (n=1). Results showed mixed engagement with the intervention (n=3); favorable user perspectives, including acceptability and cultural appropriateness (n=6); and mixed outcomes for clinical effectiveness (n=10). A diverse range of risks of bias were identified, the most common of which included a lack of clarity about allocation and blinding protocols and group treatment for randomized controlled trials and a lack of control group and single outcome measures for quasi-experimental designs. First Nations expertise informed all mHealth studies, through authorship (n=8), affiliation with First Nations bodies (n=3), participatory study design (n=5), First Nations reference groups (n=5), or a combination of these.

**Conclusions:**

mHealth modalities, including SMS and apps, appear favorable for delivery of health interventions with First Nations populations, particularly in the area of mental health and suicide prevention. Importantly, First Nations expertise was strongly embedded within the studies, augmenting favorable use and user engagement. However, evidence of efficacy is limited.

## Introduction

Mobile phones and other wireless devices have the potential to disrupt traditional health service delivery by enabling consumers to engage with health information, comanage conditions, and gain support for health challenges [1-3]. This transformation, known as mobile health (mHealth), is underpinned by the near-ubiquitous presence and functionality of mobile devices, placing health-promoting initiatives in the hands of consumers [4,5]. Furthermore, mHealth may extend equitable health access to underserved populations, such as First Nations Peoples [6,7]. Research has begun to investigate the potential role of mHealth in responding to the health disparities which continue for First Nations people [8-13]. Jones et al report that alongside other technologies, mHealth is being adopted and adapted for cultural relevance within First Nations communities where meaningful participatory approaches are applied [14,15]. Enthusiasm and engagement with the use of mobile devices in health interventions with a variety of health challenges such as mental health and hepatitis B have been reported [14,16]. However, some concerns with regard to accessibility have been noted. Primarily, a digital divide exists through lack of reliable access to hardware, connectivity services and infrastructure depending on location [14,17], and mobile devices are often a shared resource within the community, resulting in intermittent access for some individuals [18,19].

To date, few reviews with a focus on mHealth with First Nations populations have been published. A scoping review by Brusse et al [15] identified peer-reviewed evidence within both a global, and Australia’s First Nations context, for social media and mobile apps as health promoting modalities for smoking cessation, otitis media, and sexual health. Jones et al [14] conducted a critical review using Indigenous research methodology on the development and use of assistive technologies (ie, assistive devices, mHealth, and eHealth) in First Nations populations. These reviews provide valuable insights into the importance of user development, the paradox of a digital divide yet the high adoption of mobile technology, and use of social media for health interventions. However, there remains a need to synthesize the characteristics, quality, and outcomes of peer-reviewed experimental mHealth interventions with First Nations populations, across a wider range of health topics. Consideration of how mHealth research has engaged First Nations expertise is also needed to inform future research aiming to deliver health information and support to First Nations peoples.

The aim of this review was to systematically review the evidence for the use of mHealth interventions to address health challenges with First Nations populations. Specifically, the review aimed to identify characteristics of mHealth interventions (eg, study locations, health topic, and modality) evaluated with First Nations populations and to summarize the outcomes reported in relation to intervention use, user perspectives including cultural responsiveness, and clinical effectiveness. In addition, the review sought to identify the presence of First Nations expertise in the design and delivery of mHealth interventions with First Nations populations.

## Methods

A systematic review of the peer-reviewed literature was conducted. A protocol outlining the methods of this systematic review were registered with the International Prospective Register of Systematic Reviews (PROSPERO, CRD42019123276) and published on their website [20]. The review is reported in accordance with the Preferred Reporting Items for Systematic Reviews and Meta-Analyses (PRISMA) statement [21].

### Eligibility Criteria

Eligible studies were experimental or quasi-experimental study designs using quantitative, qualitative, or mixed methods that focused on the use of mHealth to address a health challenge with First Nations populations. For the purpose of this review, mHealth was defined as per the World Health Organization [3], as the delivery of health interventions via wireless devices (ie, mobile phone, smartphone, cell phone, tablet, personal digital assistant, or pocket personal computer) using wireless modalities (ie, app, short messaging service (SMS), multimedia messaging service (MMS), voice messages, email, and program or website designed for mobile or tablet use). Studies were excluded if they involved mHealth interventions delivered via websites designed for nonmobile devices, interventions with specific equipment (eg, telehealth systems), and intelligent assistive technology using cognitive devices, physiologic or environmental sensors, and tracking capabilities. Studies reporting at least 1 of the following outcomes were included: (1) use of the mHealth intervention (eg, clicks, SMS replies, and logins); (2) user perspectives of the mHealth intervention (eg, acceptability, barriers, or enablers to its use, functionality, and cultural responsiveness); and (3) clinical effectiveness (eg, impact on screening, clinic attendance, medication compliance, and knowledge). Eligible papers were full text, published in English and reported outcomes of studies conducted in Australia, Canada, New Zealand, and the United States. These countries were the focus of this review given the similarities in colonialist history and policies with intergenerational impacts for First Nations people [14,22]. Studies were included if participants from these 4 countries were either First Nations (or if part of a multicultural sample, First Nations results were specifically reported) or parents or service providers (either indigenous or nonindigenous) of First Nations people. Research protocols, editorials, abstracts, reviews, and case reports were excluded.

### Search Strategy

In October 2018, the following databases were searched systematically and without any limits to publication date: Cochrane Library, EMBASE, CINAHL, MEDLINE via PubMed, Scopus, Web of Science, and PsycINFO. A comprehensive list of search terms and strings for the key themes of *mHealth* and *First Nations or Indigenous* was developed with librarian assistance. Search strings included proximity operators, truncation, and phrase searching to explore possible iterations of both themes. To ensure capture of all relevant materials within the Australian Indigenous context, 3 additional Australia-specific searches were conducted in PubMed, CINAHL, and PsycINFO following recommendations of the Lowitja Institute [23]. A sample of search strings is available in Multimedia Appendix 1.

### Study Selection

Selection of relevant scientific papers followed the PRISMA flowchart [21]. Citations and abstracts were exported, and duplicates were removed. Screening of both the title and abstract and then remaining full-text papers was conducted independently by 2 of 3 authors at each stage (GH, DL, and MN), with discussions to reach consensus. Reasons for exclusion of full-text papers were noted. Manual searches of reference lists were conducted to identify any further studies.

### Data Extraction and Risk of Bias

Data extracted from each full-text paper included the following: title, publication year, authors, country and region, conflicts of interest, database, study aims, intervention name, health challenge addressed, study design, setting, intervention focus, intervention modality, intervention content, control, key outcome measures and instruments, outcome assessment timing, outcome assessment modality, participants, recruitment method, response rate, loss to follow-up, and other notes. Intervention outcomes were noted under 3 main headings: use, user perspectives including perceived cultural responsiveness, and clinical effectiveness.

The extent to which First Nations expertise was present within or sought by research teams in the studies was assessed via data drawn from full-text papers using authorship lists or public author profiles online. Authors’ statements regarding their identity or heritage were noted. Where such statements were unavailable, First Nations authorship was noted as *unknown*. Affiliations with First Nations bodies were recorded from full-text papers and Web-based profiles where available. Study designs and methods were reviewed to identify the use of participatory design principles or involvement of First Nations stakeholders in the intervention development or study conduct.

Risk of bias was assessed for each study using the relevant Joanna Briggs Institute (JBI) Critical Appraisal Checklist. This checklist assesses the methodological quality of a study and the extent to which the possibility of bias has been addressed in study design, conduct, and analysis [24,25]. Two authors (GH and LC) independently rated the risk of a range of biases for each included study as *yes*, *no*, *unclear*, or *not applicable* and tallied the number of *yes* responses, with consensus achieved via discussion. To enable comparison across studies using checklists with different numbers of items (9 items for quasi-experimental studies and 13 items for randomized controlled trials), a percentage was calculated. Level of Evidence (LoE) for effectiveness was also determined via a JBI tool that provides a rank of study designs “based on the likely best available evidence” [25] (p. 4). Study designs were compared with the 5 overall levels (*Level 1: Experimental* through to *Level 5: Expert Opinion and Bench Research*), with the specific level selected from possible subsets.

### Data Synthesis

A narrative approach was used for data synthesis because of the mix of study designs and research approaches. Data were summarized under 7 headings as follows: (1) study characteristics, (2) design and content of mHealth Interventions, (3) risk of bias, (4) First Nations expertise, (5) intervention use, (6) user perspectives, and (7) clinical effectiveness .

## Results

**Figure 1:**
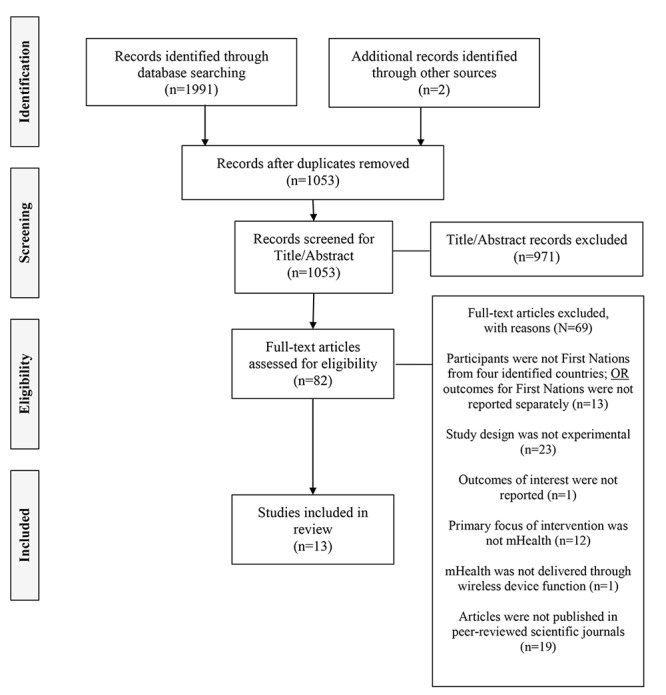
Flowchart of identification, screening, eligibility, and inclusion of scientific papers. mHealth: mobile health.

From database searches, 1051 unique records were identified; 2 additional papers were located by manual searches, with a total of 1053 papers screened at the title and abstract level. After exclusion of 971 papers, 82 full-text papers were screened. A further 69 papers were excluded, most commonly due to a nonexperimental design (n=23) or absence of full text (n=19); 13 studies were included in the narrative synthesis (Figure 1).

### Study Characteristics

All 13 studies included in this review were published between 2005 and 2018 (Multimedia Appendix 2): 5 studies were randomized controlled trials, where control groups were waitlisted for intervention (n=1) [26], received usual care (n=1) [27], had limited contact via SMS (n=2) [28,29], or received a combination of SMS, a paper resource, and usual care (n=1) [30]; 8 studies were single-arm quasi-experimental designs with a mix of qualitative and quantitative outcomes. Most studies were conducted in Australia (n=9), others in New Zealand (n=2) and the United States (n=2). No eligible studies were identified from Canada. All mHealth interventions were carried out in community settings or a combination of community and clinic and hospital settings. Participant demographics across the studies varied owing to the nature of the interventions. Adolescents and younger adults (15-30 years) were participants in 3 interventions focused on sexual health [31], new fatherhood [32], and suicide [26], whereas middle to older adults (40-75 years) were participants in studies relating to colorectal screening [27] and heart failure patient support [33]. In total, 2 studies recruited parents of younger children for interventions around child health [28,34], while other studies sought a wider sample of age for the intervention [29,30] or did not state participants’ age (n=2) [35,36]; 8 studies included a higher proportion of female participants, while 2 studies had more males and 3 did not report gender. Most studies identified participants as either First Nations (n=6) [27,31-33,36,37] or service providers to First Nations clients, or parents and carers of First Nations children (n=4) [28,34,35,38]. In studies where participants were not solely First Nations (n=3), specific outcomes were reported for First Nations people [26,29,30].

### Design and Content of mHealth Interventions

A wide range of mHealth designs, content, and intensities were used in the included studies (Multimedia Appendix 2). A total of 5 studies addressed mental health and suicide, while single studies focused on postpartum blood glucose screening, smoking, heart failure, colorectal cancer screening, chronic otitis media, hazardous drinking, infant feeding, and sexual health. Interventions were aimed at risk reduction, prevention or screening (n=7) [26,27,29-32,34], treatment and disease management (n=3) [28,33,36], or targeted multiple disease phases (n=3) [35,37,38]. The most common intervention modality was SMS (n=5) [27,29-32], followed by mobile apps (n=4) [26,35,37,38], MMS and SMS (n=1) [28], and via tablet software (not an app) (n=1) [33]. Two interventions used a combination of SMS and mobile phone or tablet apps [34,36].

#### Interventions Using Messaging (Short Message Service and Multimedia Message Service)

A total of 3 studies combined educational and supportive content via SMS to participants who were new fathers [32], wanted to quit smoking [29], or reduce their hazardous drinking levels [30]. Studies used SMS to prompt participants to attend medical clinics for colorectal cancer screening [27] or to promote sexually healthy behaviors with young Native Americans and American Indians [31]. A combination of education content and prompts for clinic appointments via SMS and MMS was used in 1 study to address chronic otitis media in children [28].

#### Interventions Using Apps

The *AIMhi Stay Strong* and *iBobbly* contained visual representations, voiceovers, action-based content, and goal setting to build mental health and reduce risk of suicide. These 2 apps were used in interventions with service providers and community members in 4 studies either separately or in combination [26,35,37,38]. One study used a tablet-delivered program for education with heart failure patients [33].

#### Other Interventions

In Australia, infant feeding knowledge was shared with parents of Aboriginal children, primarily via app or a website and SMS version for parents without the app [34]. Another study to enhance blood glucose testing following diabetes in pregnancy used several contact methods with Aboriginal women including SMS, social media, email, mobile phone calls, and face-to-face meetings [36].

Across studies, intervention modality was not closely associated with the intensity of participant contact. In the early phase of a smoking intervention using SMS, participants were contacted up to 5 times per day [29], whereas a study using SMS prompts for cancer screening contacted participants 3 times (or less) over a 3-month period [27]. Interventions using apps were generally self-guided and ranged from use during a single session [37,38] to use in multiple sessions over a longer period. For example, participants in the *iBobbly* app trial were encouraged to regularly use the app over 6 weeks to progress through modules and self-assessments.

### Risk of Bias

Using the JBI tool, LoE of the 13 studies were determined as either Level 1.c—Experimental, randomized controlled trial or Level 2.d—Quasi-experimental, Pretest—Posttest or retrospective control group study [25]. There was variability in the quality of the 5 randomized controlled trials (JBI Level 1.c), with a median quality score of 7.0 and a large range (6-11) out of 13 criteria to address risk of bias (Table 1). The 8 quasi-experimental studies (JBI Level 2.d) had a median quality score of 3.0 (range 2-6) out of 9 criteria for managing risk of bias. Once converted to percentages to allow comparison across all study designs, the risk of bias score varied across studies from 22% through to 85%. The randomized controlled trials met a higher number of quality criteria, and thus exhibited a higher mean percentage of 62%, whereas the quasi-experimental studies met fewer criteria with a mean percentage of 34%. The most common risks of bias identified in the randomized controlled trials were lack of clarity in allocation and or blinding protocols (n=5) [26-30] and absence of reliable outcome measures (n=3) [26,28,29]. Risk of bias commonly identified in the quasi-experimental studies were absence of control group (n=8) [31-38] and lack of clarity about treatment or care other than the intervention (n=7) [32-38].

A majority of the included studies (n=8) were subject to loss to follow-up. Studies acknowledged these modest (5%-12%) [26,27,29,33] or greater losses to follow-up (19%-49%) [28,30,31,36] and where appropriate, the losses to follow-up were largely accounted for through intention-to-treat analysis.

### First Nations Expertise

A notable level of First Nations expertise was embedded in the mHealth studies through authorship, consultation, and research methodology (Table 1). A variety of resources were used to identify First Nations authorship including the following: full-text research papers, author profiles, or other Web-based sources [39-50]. The majority of studies (n=8), included at least 1 author who identified as First Nations in the country in which the study was conducted [27,29,30,32,33,36-38]. A total of 3 research teams based in Australia [26,34] and the United States [31] had close affiliations with First Nations bodies, some of which served as a source for participant recruitment. First Nations expertise was also sought through participatory design principles in 5 research studies [28,31-33,35] or through key reference groups composed of First Nations stakeholders (n=5) [26,27,33,35,37].

Cultural expertise was used to ensure appropriate language, imagery, and literacy from health interventions. In 3 studies, the importance of images, design, and audio were highlighted to ensure authentic representation of First Nations identity and assist users who face literacy challenges [26,33,38]. For example, participants using a mental health app highlighted the relevance of imagery given the First Nations significance of art for knowledge transfer throughout generations [35]. Language was a central reference point in 8 studies with consideration of word content for literacy key words, cultural relevance, and local dialects [27-33,38]. Consultation with First Nations stakeholders regarding cultural relevancy helped tailor SMS content for young Aboriginal fathers in Australia [32] but did not result in local language use for SMS prompts for colorectal cancer screening in Alaska [27]. Content of mHealth interventions which drew on cultural assets such as family and relationships, and a strength-based approach to First Nations health were noted in 3 studies [29,35,38], including the *AIMhi Stay Strong App* [38].

### Intervention Use

Three studies reported participant use of the mHealth interventions with mixed results (Table 2) [26,32,36]. In a randomized controlled trial of a self-guided mental health app (*iBobbly*), 85% of participants with data available completed all 6 activities during the 6-week trial; however, use data was only available for 66% of trial participants [26]. A quasi-experimental study investigating the use of SMS to support young Aboriginal fathers in Australia also reported use by the majority of participants, with more than half clicking the link provided in 1 particular SMS (56%). The overall response rate to mood-tracking messages was 75% (106/141) [32]. In contrast, a quasi-experimental study [36] reported low response rates to clinic appointment prompts sent by SMS, phone call, email, and social media via mobile app. Although participants had nominated mobile phones as their preferred contact method, only 14% of contact attempts through the various mHealth modalities (including only 1 of 29 SMS) received a response.

**Table 1:** First Nations expertise within mobile health studies.

Study design and Study		First Nations authorship	Affiliation with First Nations bodies	Participatory design principles	First Nations reference group
**Randomized controlled trial**					
	Bramley et al [29], smoking cessation	Yes	Unknown	Unknown	Unknown
	Muller et al [27], colorectal cancer screening	Yes	Unknown	Unknown	Yes
	Phillips et al [28], chronic otitis media	Unknown	Unknown	Yes	Unknown
	Sharpe et al [30], hazardous drinking levels	Yes	Unknown	Unknown	Unknown
	Tighe et al [26], suicide risk	Unknown	Yes	Unknown	Yes
**Single-arm pre-post quantitative**					
	Yao et al [31], sexual health	Unknown	Yes	Yes	Unknown
**Single-arm pre-post mixed**					
	Clarke et al [33], heart failure	Yes	Unknown	Yes	Yes
	Dingwall et al [38], mental health	Yes	Unknown	Unknown	Unknown
**Single-arm postquantitative**					
	Kirkham et al [36], postpartum blood glucose testing	Yes	Unknown	Unknown	Unknown
	Fletcher et al [32], new fatherhood	Yes	Unknown	Yes	Unknown
**Single-arm postqualitative**					
	Dingwall et al [35], mental health	Unknown	Unknown	Yes	Yes
	Houston et al [34], infant feeding	Unknown	Yes	Unknown	Unknown
	Povey et al [37], mental health and suicide risk	Yes	Unknown	Unknown	Yes

**Table 2:** Intervention use reported across included studies.

Study design and study		Measures of intervention use; follow-up timing	Key findings
**Randomized controlled trial**			
	Tighe et al [26], suicide risk	App activity completion (total of 6); during intervention over 6 wks^a^	Participant completion: 6 activities=85% (34/40); 5 activities=2% (1/40); 2 activities=13% (5/40)
**Single-arm postquantitative**			
	Fletcher et al [32], new fatherhood	Click rates for SMS^b^ links, response to SMS (mood trackers); during intervention over 6 wks	Participant click rates: 56% *Routines: Aboriginal and Torres Strait Islander Parents*, 41% *Baby Talk*, 4% *Crying*, 0% *Bonding for Dads* and *Postnatal depression* *and* *women*. Participant response to mood trackers: 90% to at least 1×SMS, 75% to total SMS during trial (106/141)
	Kirkham et al [36], postpartum blood glucose testing	Message response via mHealth (incl. SMS, phone call, email, and social media); during intervention over 24 m^c^	14% (18/252) total responses via mHealth, including 3% response to SMS (1/29)

^a^wk(s): week(s).

^b^SMS: short messaging service.

^c^m: month.

**Table 3:** User perspectives regarding the mobile health interventions.

Study design and study		Construct measures; follow-up timing	Key findings
**Randomized controlled trial**			
	Phillips et al, [28] chronic otitis media and ear perforation, N=53	Willingness to receive future child health SMS^a^ and MMS^b^ and feedback (via survey) 6 wks^c^ from baseline	Willingness: Yes=76% (37/49), no significant difference between intervention and control groups, preference for SMS clinic prompt versus MMS=33% (3/9). Other feedback: overall interest and appreciation for message content, many shared videos
**Single-arm pre-post mixed**			
	Clarke et al, [33] heart failure, N=5	Mean participant satisfaction score (via survey), other feedback (via interview), Single use (1 hr^d^)	Satisfaction mean score=4.15/5. Other feedback: Acceptance: *I liked it all*, the teaching tool was *good*. Comprehension: *no big words*, *seeing that really did make me realize*. Impact: *This is something I will never forget*. Usability: some frustration with touchscreen (1/5)
	Dingwall et al, [38] mental health, N=130	Participant feedback (via open-ended survey), single use (1 day)	Challenge to implementation: Technology availability (access to iPads and WiFi). Support for implementation: Practice
**Single-arm postqualitative**			
	Dingwall et al, [35] mental health, N=15	Acceptability, feasibility, applicability of app (via interviews and thematic analysis), 1 m^e^	Positive responses: Acceptability: visual appeal, ease of use, cultural relevance, innovative format, etc. Building relationships: help therapeutic relationships, shift power imbalance, build client ownership, etc. Applicability: broad suitability for age and regions, mixed views concerning digital literacy. Constructive feedback: Constraints to implementation: technology availability, time, staff, local language. Integration with systems: data merges with existing records. Training recommended for content and processes
	Houston et al, [34] infant feeding, N=10	Viability, cultural appropriateness, design and language, target audience of app (via interviews and thematic analysis), 6 wks	Positive responses to viability: Familiar technology and source of information, app better than website, language, and content- trustworthy, useful, helpful, consistent and reassuring, single source of info was valuable versus searching websites. Mixed views on usability: Some frustrations with app functionality, technical difficulties, and amount of content but did not prevent use of app. Mixed views on cultural appropriateness: Service providers felt more cultural responsiveness was needed; however, parents did not. Suggestions to improve relevance: integration of personal stories, content for other carers, and guidance from key community member. Mixed views on design: Additional images, colors and art work was desired
	Povey et al, [37] mental health and suicide risk, N=9	Acceptability of *AIMhi Stay Strong App* and *iBobbly* assessed via interviews and thematic analysis, single use, same day	Overall enthusiasm and optimism for app concepts and progressive support for mental health. Influencers of acceptability: Person: illness, history, tech competence, literacy, and local language; Environment: community awareness, stigma, and support; App: content, graphics, animation, ease of use, access, security, and information sharing

^a^SMS: short messaging service.

^b^MMS: multimedia messaging service.

^c^wk(s): week(s).

^d^hr: hour.

^e^m: month.

### User Perspectives

A total of 6 studies reported participants’ views of acceptability, cultural safety, or relevance for mHealth to deliver health interventions (Table 3). In that, 1 study used phone messaging [28] and 5 studies involved apps (Table 3) [33-35,37,38]. User perspectives were reported using quantitative outcomes from surveys or interviews [28,33] and qualitative data from interviews and focus groups [34,35,37].

User perspectives on phone messaging: All participants of a randomized controlled trial reported SMS and MMS communications as culturally appropriate [28]. The majority (76%) indicated they would be happy to receive health messages in the future, although 3 of 9 participants preferred SMS clinic reminders to educational MMS [28].

User perspectives on tablet or phone apps: An intervention delivered via tablet software in a quasi-experimental study employed a survey with a 5-point Likert scale to assess aspects of navigation and usability. Participants responded to positively phrased statements with 1=strongly disagree to 5=strongly agree, and where statements were phrased negatively, scores were reversed [33]. The mean participant satisfaction score for the heart failure education program was calculated at 4.15/5 (n=5). Participants also answered open-ended questions relating to acceptance, comprehension, and impact of the app. User satisfaction scores and open-ended feedback indicated user comprehension, positive impact, and overall *acceptance* of the program and its modality, despite being the first occasion each participant had used a tablet [33].

The other 3 quasi-experimental studies reported qualitative data for user perspectives from interviews and focus groups. Mental health service providers for Aboriginal and Torres Strait Islander people expressed positive feedback for the *AIMhi Stay Strong App* through themes of acceptability, building relationships, and applicability [35]. Constructive feedback regarding barriers to practical use of the app were also recorded. Povey et al [37] gathered user perspective data on factors that would impact acceptability of the *AIMhi Stay Strong* and *iBobbly* apps. Focus group themes included characteristics of the person, the environment, and the app. Participants expressed enthusiasm for the progressive nature of the apps in supporting Australia’s First Peoples with mental health challenges. Local language was referred to as a key element for acceptability and transferability [35,37,38].

Qualitative data from parents who used an infant feeding app indicated the intervention was well received and culturally appropriate, despite no cultural tailoring [34]. In contrast, service providers (staff) felt the lack of cultural consideration could be a barrier to use. However, parent participants viewed the app as a viable delivery method for quality content. Parent participants also expressed their familiarity with mobile phone technology and a preference for the app versus a website. Some technology frustrations were experienced by participants; however, this did not inhibit their use of the app. Parents suggested embedding personal stories in the app to further connect them with program content and lift cultural relevance [34].

### Clinical Effectiveness

A total of 10 studies reported data relating to the impact of an mHealth intervention, although the outcomes varied significantly owing to the nature of interventions, study designs, and outcome measures (Table 4). Of the 10 studies, 6 were delivered using phone messaging (SMS and MMS), including 4 randomized controlled trials [27-30] and 2 quasi-experimental studies [31,36]. The remaining 4 studies involved apps, tested through randomized controlled trial (n=1) [26] or quasi-experimental designs (n=3) [33,34,38].

#### Clinical Effectiveness for Phone Messaging

The 4 randomized controlled trials of phone messaging interventions assessed diverse health outcomes [27-30]; 3 studies reported statistically significant outcomes. Bramley et al [29] reported the relative risk of smoking cessation for 
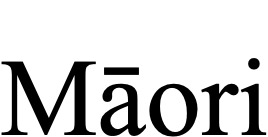
 participants who received an SMS intervention was 2.34 at 6 weeks (95% CI 1.44 to 3.79; *P*<.001) compared with controls. Self-report quit rates at 12 and 26 weeks remained high but were not statistically significant compared with controls [29]. Muller et al [27] reported that the hazard ratio of colorectal cancer screening was significantly higher after an SMS intervention compared with control (1.30 hazard ratio; 95% CI 1.04 to 1.62; *P*=.02). However, when stratified by age group, intervention participants’ screening remained higher than control, but were not statistically significant [27]. Hazardous drinking scores were significantly improved following an SMS intervention compared with control participants at 3, 6, and 9 months in a study by Sharpe et al [30]. Following discharge from hospital, intervention participants reported significantly lower scores compared with controls on the Alcohol Use Disorders Identification Test—Consumption at 3 months (−0.322; 95% CI −0.636 to −0.008), 6 months (−0.296; 95% CI −0.474 to −0.118), and 9 months (−0.260; 95% CI −0.463 to −0.057) [30]. While Alcohol Use Disorders Identification Test—Consumption scores were not reported separately for 
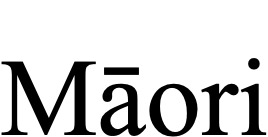
 participants, preplanned secondary analysis revealed that the intervention was effective regardless of ethnicity.

In contrast, Phillips et al [28] reported no significant difference for impact of an SMS and MMS intervention on the primary outcome of clinic attendance. No mean difference was recorded for clinic visits per child for any reason (−0.1; 95% CI −1.1 to 0.9; *P*=.90) or clinic visit per child for ear health (−0.3; 95% CI −0.8 to 0.2; *P*=.50). In addition, no significant differences were observed for the secondary outcome of diagnoses [28].

Two quasi-experimental studies involving phone messaging reported mixed clinical outcomes related to sexual health [31] and postpartum glucose testing [36]. Yao et al reported significant improvements from baseline to 1 week post-SMS for condom use and sexually transmitted infection (STI)/HIV screening for the following: (1) attitude to condom use (odds ratio [OR] 3.25; 95% CI 1.44 to 7.35; *P*=.005); (2) condom use behavior (OR 2.43; 95% CI 1.15 to 5.13; *P*=.02); and (3) intention to undergo STI/HIV testing (OR 2.46; 95% CI 1.42 to 4.26; *P*=.01). These positive outcomes were sustained at 3 months despite nonsignificant shifts in other sexual health constructs (knowledge, intention, and self-efficacy), and despite notable loss to follow-up in surveys [31]. Kirkham et al reported a lack of significant difference in completion of postpartum glucose testing for women (n=52) who were *responders* to clinic reminder messages (55%) versus those who were not (43%). Although some differences were observed in completion of certain tests for *responders* versus *nonresponders*, conclusive statements are impacted by loss to follow-up (n=10/52) and the small sample size. Furthermore, this study used other modes of communication than mHealth, and contact method was not reported against participation [36].

**Table 4:** Clinical effectiveness of mobile health interventions.

Study design, study, and sample size		Construct measures; follow-up timing	Key findings
**Randomized controlled trials**			
	Bramley et al [29], smoking cessation, N=355^a^	RR^b^ self-report smoking cessation at 6 wks^c^, 12 wks, 26 wks	2.34 RR versus control (6 wk) *P*<.001, 1.37 RR versus control (12 wk) *P*=.11, 1.17 RR versus control (26 wk) *P*=.46
	Sharpe et al [30], hazardous drinking, N=126	Mean difference in hazardous drinking scores (AUDIT-C)^d^ at 3 m^e^, 6 m, 12 m	−0.322 versus control (3 m) *P*=.04, −0.296 versus control (6 m) *P*=.002, −0.260 versus control (12 m) *P*=.002
	Muller et al [27], colorectal cancer screening, N=2386	HR^f^ of cancer screening in medical register at 6 m	1.30 HR versus control (all ages) *P*=.02, 1.42 HR versus control (50-75 years) *P*=.07, 1.24 HR versus control (40-49 years) *P*=.12
	Phillips et al [28], clinic attendance and chronic otitis media, N=53	Mean difference in clinic attendance, diagnosis of chronic otitis media, or ear perforation at 6 wks	Clinic attendance: −0.1 mean difference clinic visits versus control *P*=.90, −0.3 mean difference clinic visit ear health versus control *P*=.50; Risk difference for diagnosis: no perforation 6% (−10, 20) *P*=.60, acute otitis media with perforation N/A, dry perforation −5% (−30, 20) *P*=.70, chronic suppurative otitis media −1% (−30, 30) *P*>.99
	Tighe et al [26], suicide risk, N=61	Effect size (Cohen *d*) for suicidality (DSI-SS^g^), impulsivity (BIS^h^), depression (PHQ-9^i^), psychological distress (K-10^j^) at 6 wks	DSI-SS=0.00 (95% CI −0.51 to 0.51) *P*=.30^k^, BIS=not reported, PHQ-9=0.71 (95% CI 0.17 to 1.23) *P*=.007, K-10=0.65 (95% CI 0.12 to 1.17) *P*=.02
**Single-arm pre-post quantitative**			
	Yao et al [31], sexual health, N=408	OR^l^ condom use at 1 wk and 3 m: (1) knowledge, (2) attitude, (3) intention—partner and self, (4) behavior; OR test STI^m^/HIV at 1 wk and 3 m: (5) self-efficacy make appt, (6) self-efficacy speak to HCP^n^, (7) attitude, (8) intention	OR at 1 wk: (1) 1.15, *P*=.70; (2) 3.25, *P*=.005; (3) partner 1.33, *P*=.30; (3) self 0.97, *P*=.92; (4) 2.43, *P*=.02; (5) 1.63, *P*=.50; (6) 1.01, *P*=.99; (7) 1.11, *P*=.81; (8) 2.46, *P<*.001. OR at 3 m: (1) 1.30, *P*=.48, (2) 3.93, *P*=.002; (3) partner 1.07, *P*=.82; (3) self 0.87, *P*=.61; (4) 2.60, *P*=.02; (5) 6.77, *P*=.10; (6) 1.12, *P*=.75; (7) 0.56, *P*=.15; (8) 2.64, *P*<.001
**Single-arm postquantitative**			
	Kirkham et al [36], postpartum blood glucose screening, N=52	Attendance postpartum glucose testing (random glucose, fasting glucose, OGTT^o^ and/or HBA_1C_^p^) for message responders. Follow-up timing unclear	55% (12/22) responders any test versus 43% (13/30) nonresponders (nonsignificant) 32% (7/22) responders for OGTT versus 7% (2/30) nonresponders (nonsignificant)
**Single-arm pre-post mixed methods**			
	Clark et al [33], heart failure, N=5	Heart failure knowledge via questionnaire, self-care indicators (maintenance, confidence, and management) via SCHFI^q^ at same day	Knowledge (/20): mean score +2.0. SCHFI indicators (/100): Maintenance: mean score +16.7 (SD 25.2), Confidence: mean score + 44.4 (SD 20.8), Self-management: mean score +1.0 (SD 18.2)
	Dingwall et al [38], mental health, N=138	Pre-post knowledge and confidence (12 items) for *AIMhi Stay Strong App* via visual analog scales and open-ended questions, likelihood of using *AIMhi Stay Strong App* in next 6 m at same day	Total sample: Increase in mean scores across 10 items postintervention *P*≤.01. Subgroups: No difference between groups for mean scores across 10 items postintervention; significant difference between groups with First Nations participants’ mean scores lower for *confidence in use of other computers*, *P*<.01. Intention to use app in next 6 m: 83% total sample
**Single-arm postqualitative**			
	Houston et al [34], infant feeding, N=10	Self-report infant feeding knowledge and practice via interview at 6 wks	Self-report increases in infant feeding knowledge that informed feeding practices

^a^Number of First Nations participants from total sample.

^b^RR: relative risk.

^c^wk(s): week(s).

^d^AUDIT-C: Alcohol Use Disorders Identification Test—Consumption.

^e^m: month.

^f^HR: hazard ratio.

^g^DSI-SS: Depressive Symptom Inventory—Suicidality Subscale.

^h^BIS: Barratt Impulsivity Scale.

^i^PHQ-9: personal health questionnaire-9.

^j^K-10: Kessler-10.

^k^*P* value for the interaction of intervention arm by time (preintervention vs postintervention).

^l^OR: odds ratio.

^m^STI: sexually transmitted infection.

^n^HCP: health care professional.

^o^GTT: oral glucose tolerance test.

^p^HBA_1c_: hemoglobin A_1c_.

^q^SCHFI: self-care heart failure inventory.

#### Clinical Effectiveness for Mobile Apps

A randomized controlled trial reported clinical effectiveness measures for the self-guided *iBobbly* app which includes the following: suicidality (Depressive Symptom Inventory—Suicidality Subscale [DSI-SS]), impulsivity (Barratt Impulsivity Scale), depression (Personal Health Questionnaire), and psychological distress (Kessler-10). Tighe et al [26] reported no significant reduction in the primary outcome of suicidal ideation between intervention and waitlist groups at 6 weeks (mean DSI-SS scores=1.9 in both groups), despite a reduction in pre-post mean DSI-SS scores for the intervention arm (pre=2.7, SD 2.2; post=1.9, SD 2.1). Secondary outcomes for intervention versus waitlist groups at 6 weeks were mixed, with no significant change in impulsivity, yet significant improvements in scores for depression (Personal Health Questionnaire-9=8.9, SD 5.4 vs 12.8, SD 5.5) and psychological distress (Kessler-10=22.7, SD 7.4 vs 27.9, SD 8.0) [26].

Three quasi-experimental studies reported outcomes for participant knowledge following interventions using phone or tablet-based apps or programs. Houston et al [34] supported parents via an infant feeding app over 6 weeks. Reports included an increase in knowledge and alterations to their infant feeding practices. Service providers trained in use of a mental health app over 1 day by Dingwall et al showed significant increases in knowledge and confidence across 10 of 11 items assessed in a questionnaire with visual analogue scales [38]. Similarly, Clark et al [33] reported participants’ mean increase of 2 points on heart failure knowledge scores out of a possible 20 points (9.6, SD 1.3 to 11.6, SD 1.9) following 1-h use of a tablet-based education program. Increases in mean Self-Care Heart Failure Index scores were also reported. However, the results should be interpreted with caution as they were accompanied by incomplete Self-Care Heart Failure Index surveys (n=2) and high standard deviations, reflecting the small sample (n=5).

## Discussion

### Principal Findings

#### Mobile Health Research Characteristics—Studies and mHealth Interventions

There is limited reporting on the characteristics of mHealth interventions evaluated with First Nations Peoples, with 13 studies identified in this review. A number of studies were randomized controlled trials involving messaging, commonly available across devices and platforms through SMS and MMS. Previous studies suggest text messaging is efficacious, relatively easy to use and a low-cost modality [2,3,15,51]. The messaging-based interventions in this review drew on these strong characteristics to respond to health challenges with First Nations populations. Other studies used digital add-ons, including unique mobile apps or programs using mobile software. Despite their prolific presence and adoption by consumers, research into the health impact of apps lags behind that of SMS. This reflects the more recent development of this modality and subsequent recognition of a public health potential [52].

Intervention content and contact intensity varied significantly owing to the wide range of health topics addressed with First Nations populations. However, mental health and suicide prevention, particularly in the Australian context, were common themes (n=5). This is an important finding given the documented mental health challenges experienced by First Peoples of Australia, New Zealand, and the United States on account of intergenerational trauma [22]. It demonstrates that scientific research is responding to the mental and emotional challenges faced by First Nations populations [53].

Furthermore, this review noted that the diversity of First Peoples was recognized through collaborative projects, designed and trialed in local contexts, with consideration of the transferability of mHealth products rather than a one-size-fits-all approach [16,37]. Research teams were embedded with First Nations knowledge and leadership to explore the potential role of technology for health. First Nations expertise strengthened the relevance and transfer of the mHealth research and influenced participant engagement and outcomes for the interventions.

#### Mobile Health Outcomes

The diversity of mHealth aims and interventions yielded a mix of reported key outcomes across 3 domains: *use*, *user perspectives*, and *clinical effectiveness*.

First, despite participant assurance of access to or ownership of mobile devices, engagement levels with messaging and apps were varied [26,32,36]. While only 3 studies reported on this outcome, it is feasible that cultural tailoring and First Nations expertise during mHealth development enhanced the use of mHealth in 2 trials [26,32]. Authors of the study with lower use [36] acknowledged the wider social and environmental priorities that may influence engagement with an intervention. In particular, mobile devices may be viewed as a shared resource within First Nations communities [18,19,36], thus impacting the potential exposure of an mHealth intervention. Furthermore, the accuracy of data relating to phone ownership and use in First Nations communities (especially remote areas) is problematic given a digital divide shaped by location, network infrastructure, cost of devices, and network access [17,52].

Second, overall favorable user perspectives were reported for 6 Australian mHealth studies [28,33-35,37,38]. High acceptability and user satisfaction were noted, even for participants without previous experience with devices [33,37]. Navigation difficulties and technical challenges with apps were a concern for some [37], although other participants were not deterred [34]. Notably, First Nations participants reflected on the progressive nature of mHealth to support communities in novel ways [35,37]. Others resonated with emphasis on First Nations assets (family and relationships) in the programs [35] and expressed the likelihood of using an mHealth service in the future if available [28,33,38]. Interestingly, some concerns about cultural safety by service providers were not mirrored by service recipients [34,35,37,38]. The acceptability of mHealth interventions reported here is in line with descriptions by Brusse et al [15], who noted high penetration and rapid uptake of mobile interfaces such as social media, apps, and messaging. Importantly, beyond social or entertainment value, mobile devices offer significant potential for access to health support in scenarios where individuals hold concerns about face-to-face interaction. For example, tablets may facilitate help-seeking behavior for young people who experience concerns about social interaction and moral judgement when accessing mental health support [26,37,38,53,54]. This systematic review identified significant First Nations expertise that conceivably augmented favorable user perspectives of the mHealth interventions. For example, the *AIMhi Stay Strong App* has been subject to significant work on cultural feasibility, design, and usability with guidance from First Nations stakeholders, and has been met with highly positive feedback by service providers [35,37,38]. Genuine cultural consultation through collaborative research and participatory design in other studies has been associated with high consumer interest [14] and considered paramount for cultural relevance and safety [16,51].

The clinical effectiveness of the mHealth interventions with First Nations populations in this review, while mixed, were in agreement with other reports. Although considered ideal for health interventions [4,6,7], conclusive statements for the clinical efficacy of mHealth are limited. In a systematic review of systematic reviews (n=23) for mHealth research, Marcolino et al [2] reported that evidence for mHealth efficacy was limited to several chronic health interventions including asthma and smoking cessation, mostly via basic messaging functionality of phones. Authors emphasized that a majority of studies in the reviews were of low methodological quality. Many of the included reviews found either no significance or conflicting outcomes, with a need for further studies to address quality and validation of pilot work with repeat trials of longer duration. The present review included a high heterogeneity across study designs, interventions, risk of bias, and outcome measures, limiting conclusive statements around effectiveness or efficacy.

The challenges of mHealth research described by Marcolino et al [2] and the present systematic review correspond with broader public health intervention research, where evidence for causality is often constrained by sample size, time, ethical considerations, and costs. Although these limitations can result in lower levels of empirical evidence, health-promoting interventions should also be evaluated on their “completeness and transferability” (p. 125) [55] to allow authentic evidence within the complex environment in which real-world interventions are trialed. Ben-Zeev et al emphasized the challenges associated with development and trial of mHealth interventions for use “in the wild” (p. 158) [56], noting that they require realism, collaboration, and flexibility of research teams. Several recent app trials have demonstrated the research possibilities and broader significance of mHealth interventions beyond solely clinical data, with encouraging results. For example, while research reports in this review [35,37,38] for the *AIMhi Stay Strong App* would traditionally be considered low levels of evidence [55], this app is informed by research showing that motivational care planning (MCP) effectively improved measures of well-being, substance misuse, and self-management among Aboriginal and Torres Strait Islander clients with chronic mental illness [57]. The subsequent adaptation of MCP into the *AIMhi Stay Strong App* could be deemed an mHealth success story as it is now used by health and community services in Australia [58]. This app has undergone several reiterations over recent years to suit a variety of population groups and organizational needs (personal communication, J Povey, May 14, 2019). It is also currently being trialed as a well-being intervention with Aboriginal and Torres Strait Islander people with chronic kidney disease [59]. In addition, while the pilot trial of the *iBobbly* app did not find clinically significant results for suicide ideation, the significant outcomes for depression and psychological distress have justified further app development and a national randomized controlled trial to continue the important work in this area [53].

### Limitations

The ever-evolving nature and terminology of mobile health technology presents some challenges. To ensure consistent inclusion and exclusion of intervention studies, this paper adhered to the World Health Organization definition of mHealth [3], which includes text messaging, mobile apps, and other wireless modalities. Although this definition facilitates inclusion of a wide range of interventions, thus strengthening the review, it is acknowledged that some limits could have been set by the authors in an effort to manage the breadth of mHealth interventions. Publication bias may also have impacted the identification of mHealth studies with First Nations populations, whereby studies with inconclusive or negative findings may not have been published [60]. In addition, this review did not include unpublished (*gray*) literature, which may have yielded further studies examining mHealth interventions. Quality assessment was conducted using 2 JBI tools to allow risk of bias comparison across studies using differing designs, with 2 authors independently assessing each study to minimize error in the quality assessment. However, it is recognized that quality assessments may reflect limitations in reporting (eg, owing to publication space) rather than study conduct. Similarly, First Nations expertise was assessed using desktop research only. Information on authorship, key reference groups, participatory design, and affiliations was not always clear or available. The authors acknowledge that studies may have drawn on additional features of First Nations expertise.

### Conclusion

This is the first systematic review to document the characteristics, use, user perspectives, and clinical effectiveness of mHealth from peer-review scientific studies, with unrestricted health topics, where participants are First Nations people, or parents or service providers to First Peoples from Australia, New Zealand, or the United States. Encouragingly, mHealth interventions have been conducted with considerable First Nations expertise, rather than mere consultation, to guide cultural usability and meaning for community. This is a meaningful finding given that First Nations knowledge is the most valuable asset available to address challenges of First Nations health and community [61]. It is a feasible conclusion that embedding First Nations expertise into the projects helped augment favorable reports and feedback for use and user perspectives in the mHealth interventions. Difficulty remains, however, in the ability to draw conclusive statements about clinical effectiveness given the heterogeneity of mHealth interventions with First Nations populations. Clinically significant outcomes have been reported for both messaging and app modalities. While messaging appears to have a more mature evidence base, apps are acceptable, and they are being used effectively with First Nations Peoples. Trials are underway to validate previous findings of significance and to explore the transferability of mHealth across the diversity of First Nations populations. Importantly, this ongoing research, particularly in the area of mental health and suicide prevention, holds potential for significant impact with First Nations communities.

## Appendix

Multimedia Appendix 1 Sample search strategies, October 2018.

Multimedia Appendix 2 Study design, risk of bias, and mobile health (mHealth) intervention characteristics.

## Abbreviations

DSI-SS: Depressive Symptom Inventory—Suicidality Subscale
JBI: Joanna Briggs Institute
LoE: level of evidence
MCP: motivational care planning
MMS: multimedia messaging service
OR: odds ratio
PRISMA: Preferred Reporting Items for Systematic Reviews and Meta-Analyses
SMS: short messaging service
STI: sexually transmitted infection
