# Supplementary material for: Mobile Health for First Nations Populations: Systematic Review
Source: JMIR Mhealth Uhealth. 2019 Oct 7;7(10):e14877. doi: 10.2196/14877 (PMC6803895; doi:10.2196/14877)
Supplement: Multimedia Appendix 1 [file mhealth_v7i10e14877_app1.pdf]

Multimedia Appendix 1: Sample search strategies, October 2018

**A. mHealth keywords used in EMBASE**	
**Limits: English**	
‘e-mail'/exp; ‘text messaging'/exp; 'mobile device'/exp; ‘telenursing'/exp; ‘tele nurs*':ti,ab,kw; ‘telemedicine'/exp; 'tele medicine*':ti,ab,kw; tele health':ti,ab,kw; telehealth:ti,ab,kw; ‘telehealth'/exp; ‘mobile phone'/exp; 'mobilephone*':ti,ab,kw; ‘mobile phone*':ti,ab,kw; 'wirelessdevice*':ti,ab,kw; ‘wireless device*':ti,ab,kw; ‘touchscreen*':ti,ab,kw; ‘touch screen*':ti,ab,kw; 'mobile device*':ti,ab,kw; 'pocket personal computer*':ti,ab,kw; 'pocket pc':ti,ab,kw; ‘ehealth':ti,ab,kw; 'e-health':ti,ab,kw; 'e-mobile*':ti,ab,kw; ‘emobile*':ti,ab,kw; ‘mobile application'/exp; 'mobile app*':ti,ab,kw; 'portable phone*':ti,ab,kw; 'cellphone*':ti,ab,kw; ‘cellular phone*':ti,ab,kw; 'cell phone*':ti,ab,kw; 'smartphone*':ti,ab,kw; ‘smart phone*':ti,ab,kw; 'mobilehealth':ti,ab,kw; ‘mobile health':ti,ab,kw; mhealth:ti,ab,kw; m-health:ti,ab,kw; ‘m health':ti,ab,kw	**Results = 85061**
**B. Indigenous population keywords used in EMBASE**	
**Limits: English**	
(indigen*:ti,ab,kw OR 'first nation*':ti,ab,kw OR aborigin*:ti,ab,kw) AND (australia*:ti,ab,kw OR 'new zealand*':ti,ab,kw OR canad*:ti,ab,kw OR america*:ti,ab,kw); 'amer indian*':ti,ab,kw; amerindian*:ti,ab,kw; (hawaii* NEAR/3 native*):ti,ab,kw; (alaska* NEAR/3 native*):ti,ab,kw; aleut:ti,ab,kw; eskimo*:ti,ab,kw; metis:ti,ab,kw; (america* NEAR/3 samoa*):ti,ab,kw; (america* NEAR/3 native*):ti,ab,kw; 'america* indian*':ti,ab,kw; (america* NEAR/3 indian*):ti,ab,kw; koori:ti,ab,kw; tiwi:ti,ab,kw; torres strait':ti,ab,kw; inuit*:ti,ab,kw; aborigin*:ti,ab,kw; ‘first nation*':ti,ab,kw; (indigen* NEAR/5 america*):ti,ab,kw; (indigen* NEAR/3 canad*):ti,ab,kw; (indigen* NEAR/3 australia*):ti,ab,kw; indigen*:ti,ab,kw; 'native hawaiian'/exp; ‘oceanic ancestry group'/exp; ‘indigenous australian'/exp; ‘first nation'/exp; 'canadian aboriginal'/exp; 'alaska native'/exp; ‘american indian'/exp	**Results = 75063**
**C. Combined search: Results of A and B**	**Results = 465**

**A. mHealth keywords used in PUBMED**	
**Limits: English**	
Search (((((((((((((((((((((((((((((((((((m-health[Title/Abstract]) OR mhealth[Title/Abstract]) OR m health[Title/Abstract]) OR "mobile health"[Title/Abstract]) OR "mobile phone*"[Title/Abstract]) OR mobilephone*[Title/Abstract]) OR mobilehealth[Title/Abstract]) OR "smart phone*"[Title/Abstract]) OR smartphone*[Title/Abstract]) OR "smartphone"[Title/Abstract]) OR cellphone*[Title/Abstract]) OR "cell phone*"[Title/Abstract]) OR "cellular phone*"[Title/Abstract]) OR "portable phone*"[Title/Abstract]) OR "mobile app*"[Title/Abstract]) OR "mobile applications"[Title/Abstract]) OR emobile*[Title/Abstract]) OR e-mobile*[Title/Abstract]) OR e-health[Title/Abstract]) OR ehealth[Title/Abstract]) OR "pocket pc"[Title/Abstract]) OR "pocket personal computer"[Title/Abstract]) OR "mobile device*"[Title/Abstract]) OR "touch screen*"[Title/Abstract]) OR touchscreen*[Title/Abstract]) OR "wireless device*"[Title/Abstract]) OR wirelessdevice*[Title/Abstract]) OR "electronic mail"[Title/Abstract]) OR "text messaging"[Title/Abstract]) OR "computers, handheld"[Title/Abstract]) OR telehealth[Title/Abstract]) OR "tele health"[Title/Abstract]) OR "tele medicine"[Title/Abstract]) OR "telemedicine"[Title/Abstract]) OR "tele nurs*"[Title/Abstract]) OR "telenursing"[Title/Abstract] Filters: English	**Results = 35361**
**B. Lowitja Indigenous terms (Lit.com) used in PUBMED**	**Results = 9385**
**Limits: English**	
(((((australia[mh] OR australia*[tiab]) AND (oceanic ancestry group[mh] OR aborigin*[tiab] OR indigenous[tw])) OR (torres strait* islander*[tiab])) AND medline[sb]) OR ((((au[ad] OR australia*[ad] OR australia*[tiab] OR northern territory[tiab] OR northern territory[ad] OR tasmania[tiab] OR tasmania[ad] OR new south wales[tiab] OR new south wales[ad] OR victoria[tiab] OR victoria[ad] OR queensland[tiab] OR queensland[ad]) AND (aborigin*[tiab] OR indigenous[tiab])) OR (torres strait* islander*[tiab])) NOT medline[sb]) AND English[la]) Filters: English; Field: Title/Abstract	
**C. Combined search: Results of A and B**	**Results = 82**

Authors' note: *several archaic terms were used in searches given there was no limit on dates and older studies may have employed terms no longer considered culturally appropriate. The authors recognize the need to use contemporary language and endeavor to conduct respectful communication with First Peoples internationally.*
