# Supplementary material for: Mobile Health for First Nations Populations: Systematic Review
Source: JMIR Mhealth Uhealth. 2019 Oct 7;7(10):e14877. doi: 10.2196/14877 (PMC6803895; doi:10.2196/14877)
Supplement: Multimedia Appendix 2 [file mhealth_v7i10e14877_app2.pdf]

Multimedia Appendix 2: Study design, risk of bias, and mHealth intervention characteristics.

**Study design**	**Authors**	**Location and setting**	**Sample**	**Topic and content**	**Modality and contact with participants**	**JBI,[a] LoE,[b] and score[c]**
**Randomized controlled trials**						
	Bramley et al [29]	New Zealand (national); community	N[d]=1705, 19-30 years (M[e]=22), Māori=21%, F[f]=58%	Smoking cessation: advice, support, and distraction	Intervention: SMS[g]: 5/day—early, 3/wk[h]—later; Control: SMS only	1.c, 9/13, 69%
	Muller et al [27]	Alaska (Anchorage); clinic and community	N=2386, 40-75 years, American Indian/Alaska Native=100%, F=56%	Colorectal cancer screening: confirm enrolment; screening prompts	Intervention: 1×SMS—pre, 1×SMS—1 m[i] post (unscreened), 1×SMS—2 m post (unscreened), 3×SMS over 2 m (total); Control: Usual care	1.c, 11/13, 85%
	Phillips et al [28]	Australia (Northern Territory); clinic and community	N=53, <13 years, Aboriginal =100% parent- or carer-owned mobile	Chronic otitis media: confirm enrolment; MMS[j] ear health videos; SMS personalized clinic prompts	Intervention: 1×MMS/4 days with personalized SMS for clinic prompts, 7×MMS over 6 wks (total), 2×SMS for participation; Control: 2×SMS and follow-up prompt	1.c, 7/13, 54%
	Sharpe et al [30]	New Zealand (Auckland);	N=598, 16-69 years ($\bar{x}$[k]=34),	Hazardous drinking: feedback; SMS to cut drinking levels; strategies; support	Intervention: wk 1: 4×SMS, wk 2: 3×SMS, wk 3: 3×SMS, wk 4: 3×SMS;	1.c, 6/13, 46%

		hospital and community	Māori =21.1% F=28.6%		Control: Paper brochure, usual care, 1×SMS	
	Tighe et al [26]	Australia (Kimberley), community	N=61, 18-56 years (x̄=26.3 years), Aboriginal and Torres Strait Islander=93.4% F=64%	Suicide risk: thoughts, feelings, behaviors, mindfulness, acceptance, goal setting, etc. Suicide self-assessment: functioning, state mind, and progress tracker.	Intervention: Tablet app, 6 wk exposure. Safety check with researchers at 3 and 9 wks; Control: Waitlist 6 wks	1.c, 7/13, 54%
**Single-arm pre-post quantitative**						
	Yao et al [31]	United States (national); community	N=192, 15-24 years, American Indian and Alaska Native=100%, F=77%, Straight=82% LGBT2S[l]=18%	Sexual health: knowledge, attitudes, self-efficacy, intention and behavior (condom use and STI[m]/HIV testing)	97×SMS over 9 m (total) inc. 32×SMS interv. 36×SMS survey, 29×SMS admin.	2.d, 6/9, 67%
**Single-arm pre-post mixed methods**						
	Clark et al [33]	Australia (Ipswich); community and health service	N=5, 51-71 years (x̄=61.6), Aboriginal= 100%, F=unstated, heart failure patients	Heart failure education and support: managing fluid levels and symptoms, preventing hospital admission, fluid action plan, and medical help	Tablet (PowerPoint program), single day use	2.d, 4/9, 56%

	Dingwall et al [38]	Australia (Northern Territory); community	N=130, 19-74 years ($\bar{x}$=40.1), Aboriginal and Torres Strait Islander=35%, F=70%, MH[n] service providers for Aboriginal and Torres Strait Islander clients	MH: integrate e-mental health within practice, First Nations health concepts, strengths-based care planning, risk assessment, mental state examination, and goal setting	Tablet app, single day use	2.d, 3/9, 33%
**Single-arm post quantitative**						
	Kirkham et al [36]	Australia, (Northern Territory); hospital and clinic and community	N=52, age unstated, Aboriginal=100%, F=100%, GDM[o] or T2DM[p]	Postpartum blood glucose screening: paper-based info. and reminder for testing at 6 wks, clinic prompts	Either SMS, mobile call, Facebook, email (and Face-to-Face); T2DM: 6 wks, 3 m, 6 m, 9 m, 12 m, 15 m, 18 m, 21 m, 24 m; GDM: 6 wks, 6 m, 12 m, 24 m	2.d, 5/9, 56%
	Fletcher et al [32]	Australia (regional and rural New South Wales); community	N=20, 18-25 years, Aboriginal=100%, F=0%, fathers of ≥1 child	MH and parenting support for new fathers: links to webpages and videos; SMS mood checker	25×SMS in 6 wks (~4/wk); 4-10×SMS over 6 wks for mood tracker (1-2/wk)	2.d, 3/9, 33%
**Single-arm post qualitative**						
	Dingwall et al [35]	Australia (Northern Territory);	N=15, age unstated, Aboriginal and	MH: *AIMhi Stay Strong App*: strength-based care planning,	Phone or tablet app, 1 m exposure	2.d, 2/9, 22%

		community	Torres Strait Islander=27%, F=unstated, service providers for First Nations clients	risk and mental state assessment, and goal setting.		
	Houston et al [34]	Australia (Brisbane); community and clinic	N=10, 18-35 years, F=80%, parents of Aboriginal or Torres Strait Islander child <9 m (100%)	Infant feeding: evidence-based info. tailored for feeding method and age	Phone app or SMS and website, 3×SMS or notifications per wk over 6 wks	2.d, 2/9, 22%
	Povey et al [37]	Australia (Northern Territory); community	N=9, 18-60 years ($\bar{x}$=33), Aboriginal=100%, F=67%	MH and suicide risk: *AIMhi Stay Strong app*: strength-based care planning, risk and mental state assessment, and goal setting; *iBobbly* app: thoughts, feelings, behaviors, mindfulness, acceptance, goal setting, functioning, state mind, suicidality, and progress tracker.	Tablet or phone apps, single day use	2.d, 3/9, 33%

[a]JBI: Joanna Briggs Institute.

[b]LoE: level of evidence (Level 1.c: Experimental, randomized controlled trial; Level 2.d: Quasi-experimental, Pretest–Posttest or retrospective control group study).

[c]Score: criteria met in addressing risk of bias from total possible score.

[d]N: total participants.

[e]M: median.

[f]F: female.

[g]SMS: short messaging service.

[h]wk: week.

[i]m: month.

[j]MMS: multimedia messaging service.

[k]$\bar{x}$: mean

[l]LGBT2S: Lesbian Gay Bisexual Transgender Two Spirit.

[m]STI: sexually transmitted infection.

[n]MH: mental health.

[o]GDM: gestational diabetes mellitus.

[p]T2DM: type 2 diabetes mellitus.
